# Program Bytes: Satellite Meetings, SIGs, and AKEs at ISMB 2016

**DOI:** 10.1371/journal.pcbi.1004944

**Published:** 2016-06-02

**Authors:** Christiana N. Fogg, Diane E. Kovats

**Affiliations:** 1 Freelance Science Writer, Kensington, Maryland, United States of America; 2 International Society for Computational Biology, Bethesda, Maryland, United States of America

**Figure:**
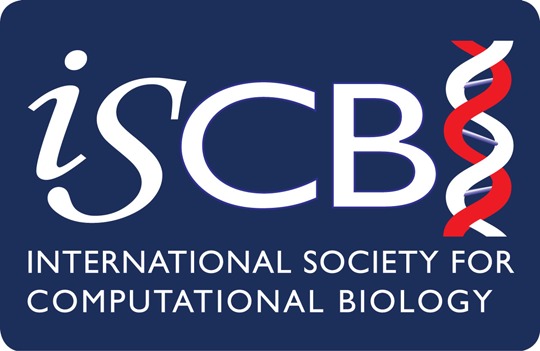


ISMB 2016 is a world-class conference for computational biologists because it also is host to several smaller meetings prior to the main ISMB meeting. As in the past, satellite meetings and special interest group meetings (SIGs) will be held on Friday, July 8th, and Saturday, July 9th, prior to the main conference at the main meeting venue, the Swan and Dolphin Hotel. Several applied knowledge exchange sessions (AKEs) have also been scheduled for Saturday and are smaller in size in order to provide a more intimate meeting environment. Check out this year’s satellite meetings, SIGs, and AKEs and be sure to register for these unique events along with your main meeting registration.

## Satellite Meetings

This year, two popular satellite meetings are convening once again for two days on Friday, July 8th, and Saturday, July 9th. 3DSig: Structural Bioinformatics and Computational Biophysics will be holding its 12th meeting and has become one of the largest meetings that brings together world-class scientists studying structural computational biology. Keynote speakers include David E. Shaw of Columbia University; Ruben Abagyan of the University of California, San Diego and Molsoft LLC; Heather Carlson of the University of Michigan; Bruce Donald of Duke University; Koby Levy of the Weizmann Institute; Rafael Najmanovich of the Université de Sherbrooke; and Shoshana Wodak of the Free University of Brussels.

The 15th annual International Conference on the Critical Assessment of Massive Data Analysis (CAMDA) is the other major satellite meeting being held on July 8th and 9th. CAMDA focuses on the analysis and integration of massive data sets, and the scientific committee of CAMDA has posted three data analysis challenges for its annual contest. Scientists present their analyses of these data set challenges and prizes are awarded for best analyses and presentations.

## SIGs

ISMB is fortunate to host numerous one- and two-day SIGs prior to the main meeting. Many of the SIGs are organized by ISCB’s Communities of Special Interest (COSIs). These SIGs span all topic areas in the field of computational biology, including bio-ontologies, high-throughput sequencing, open source bioinformatics software, data visualization, and RNA biology. There really is a SIG for everyone, and these smaller meetings provide an atmosphere conducive to scientific discussion and exchange. Two new SIGs are debuting this year: “SysMod: Computational Modeling of Biological Systems” and “TransMed 2016: Translational Medicine Informatics & Applications.” SysMod aims to close the gap between bioinformatics and systems biology modeling and will include talks on modeling metabolism and modeling biochemistry. TransMed will cover topics related to the burgeoning field of translational medicine informatics, including clinical and molecular data storage and integration infrastructure, curation and harmonization of clinical, ‘omics, and imaging data, and computational approaches for target selection and drug discovery, as well as other related topics.

## AKEs

AKEs debuted at ISMB/ECCB 2015 as a new version of workshops. These small meetings are limited in space to promote scientific discussion and exchange and are also held prior to the main ISMB meeting. This year five AKEs have been organized and they will cover a wide range of relevant and timely topics. The sessions are:

- AKES01: Clouds, Clusters, and Containers: Tools for responsible, collaborative computing
- AKES02: Community Efforts to Enable Scalable, Reproducible, and Portable Biomedical Data Analyses
- AKES03: Synthetic Biology Open Language—standards, tools and guideline on managing both the functional design and the sequence details of your experiments
- AKES04: Living on the Edge (of Translational Informatics)—Opportunities and Challenges for Integrating Bioinformatics into the Clinical Realm
- AKES05: Cytoscape 3 User Tutorial: Introduction to network visualization and analysis using Cytoscape

Make sure you consider all of these pre-meeting sessions during your conference planning and make the most of your time at ISMB 2016.

